# Preparation and Performance Analysis of Tung Cake Protein Adhesive

**DOI:** 10.3390/polym16233437

**Published:** 2024-12-07

**Authors:** Wei Wang, Ke Zheng, Wenzheng Zhao, Shenglong Zheng, Hui Wan, Jingran Gao

**Affiliations:** 1 Yunnan Provincial Key Laboratory of Wood Adhesives and Glued Products, Southwest Forestry University, Kunming 650224, China; wwrio_iy@163.com (W.W.); 19962614952@163.com (W.Z.); zzsenln@163.com (S.Z.); wanhui@swfu.edu.cn (H.W.); 2 Key Laboratory of State Forestry and Grassland Administration on Highly-Efficient Utilization of Forestry Biomass Resources in Southwest China, Southwest Forestry University, Kunming 650224, China; zhknh@126.com

**Keywords:** tung cake, protein adhesive, formaldehyde-free adhesive, alkali modification, cross-linking modification

## Abstract

Tung oil pressing generates a substantial amount of tung cake waste rich in protein, which can be used to develop a novel wood protein adhesive. This study determined the optimal alkali treatment parameters based on NaOH concentration, reaction temperature, and reaction time. Potassium permanganate (KMnO_4_) and methyl trimethoxy silane (MTMS) were then sequentially added for cross-linking modification to achieve the optimal preparation process for the tung cake protein adhesive. Bonding strength was tested on pressed boards, and various characterization techniques, including X-ray diffraction (XRD), Fourier transform infrared spectroscopy (FTIR), Thermogravimetric analysis (TG/TGA), differential scanning calorimetry (DSC), and scanning electron microscopy (SEM), were used. The results indicated the following: (1) Optimal preparation conditions: The best preparation process for the adhesive involved 30% NaOH at 50 °C for 50 min, with the addition of 12% KMnO_4_ and 6% MTMS, meeting Class II plywood standards. (2) XRD and FTIR analyses revealed that carbohydrates in the tung cake oxidized and reacted with protein amino groups. The active groups in the protein cross-linked with MTMS, forming a spatial network structure, reducing hydrophilic groups, and enhancing water resistance. (3) TG/TGA and DSC showed that the thermal stability of the modified adhesive improved, thermogravimetric loss was reduced, and curing performance was enhanced. (4) SEM verified the adhesive’s reaction mechanism, demonstrating that MTMS filled the protein structure unfolded by KMnO_4_, forming a three-dimensional network and improving bonding strength. This study successfully developed a new, formaldehyde-free, environmentally friendly tung cake protein adhesive with excellent performance.

## 1. Introduction

The wood industry plays a vital role in the economic development of many countries, with wood adhesives being central to its operations. Currently, the most widely used adhesives for wood-based panels, both domestically and internationally, are aldehyde resins derived from value-added products of the petrochemical industry [1,2]. These include phenolic resins, urea–formaldehyde resins, and melamine–formaldehyde resins. Together, these adhesives account for approximately 75% of global adhesive production, making the wood industry the largest consumer of adhesives [3]. As a result, the production and quality of adhesives are directly linked to the development level of the wood industry in various nations [4,5]. However, during the preparation, processing, and use of such adhesives and the artificial boards they are used in, formaldehyde, which is harmful to both human health and the environment, may be released [6,7,8]. This can damage the respiratory and nervous systems, leading to symptoms such as sore throat, dizziness, headaches, and memory loss. In severe cases, it may pose carcinogenic and teratogenic risks, potentially triggering cancers such as nasopharyngeal carcinoma and leukemia [9]. While polyurethane adhesives have garnered attention as a formaldehyde-free alternative, their high cost has limited their widespread adoption in the wood processing industry. Amid the growing depletion of fossil resources and the rising concern over formaldehyde pollution, environmental awareness has increased among consumers and manufacturers alike [10]. This has spurred the wood-based panel industry to focus on the development of adhesives that are low in formaldehyde or even completely formaldehyde-free [11]. In response, both research and commercial efforts have intensified to meet these environmental challenges, with a clear trend toward the use of agricultural and forestry resources in the production of sustainable and eco-friendly wood adhesives [12].

Biomass-based wood adhesives, recognized as renewable, non-toxic, and environmentally friendly, have become a major area of research both domestically and internationally [13,14]. These adhesives are derived from the chemical modification of natural polymer raw materials sourced from biomass. As green, sustainable, and renewable materials, they align with the growing demand for eco-friendly adhesives in the wood processing industry [14]. The primary raw materials for biomass-based wood adhesives include plant proteins, starches, tannins, and wood fibers. These materials offer excellent environmental benefits, contributing to sustainability [15,16]. However, the direct use of these materials as adhesives presents certain challenges, including poor water resistance, slow curing speed, and high curing temperatures, which make it difficult to meet the performance standards required for effective wood bonding [17,18,19]. To overcome these limitations, chemical modifications or combinations with synthetic polymer materials are necessary to enhance their performance and ensure compliance with industry standards [20,21,22]. In comparison to petroleum-based adhesives, plant protein adhesives offer several advantages: they feature a simpler production process, a broad range of available raw materials, low costs, and no pollution [23]. However, for effective bonding, protein adhesives must possess the right viscosity and polarity, enabling them to form directional bonds with amorphous materials like wood. Among plant proteins, soy protein has been the most extensively studied, but research on other plant proteins remains limited, and further exploration is needed. Soy protein adhesives first emerged in the 1920s, when Johnson patented the use of soy protein in adhesives [24]. In the 1950s, the development of synthetic resins derived from fossil fuels such as natural gas and oil, which offered superior adhesion and humidity resistance, led to the gradual replacement of soy protein adhesives. By the 1970s, soy protein adhesives were largely supplanted by “aldehyde” adhesives due to their inferior adhesion and water resistance properties. However, in the late 20th century, the oil crisis and rising prices of synthetic resin materials, combined with growing concerns over the harmful effects of formaldehyde and phenol emissions from synthetic adhesives, renewed interest in sustainable alternatives [25,26]. The depletion of fossil fuels and the increasing environmental awareness among both the public and regulatory bodies led to higher standards for formaldehyde emissions in composite wood products. In response, global research on protein-based adhesives, as a green and eco-friendly alternative, accelerated. Soy protein, with its abundant functional groups, high reactivity, and wide availability, has become the focus of many studies. Despite its promise, the large-scale use of soybeans for industrial applications raises concerns over the potential impact on the food supply, given that soybeans are a crucial source of protein for human nutrition [27,28]. As such, the search for alternative plant proteins to replace soy in the production of natural, sustainable biomass-based adhesives is of paramount importance. This shift will ensure that industrial applications do not compromise food security while advancing the development of environmentally friendly adhesive technologies.

The tung oil tree (*Vernicia fordii*), a deciduous species from the Euphorbiaceae family, is a unique and valuable economic forest tree in China. It is recognized as one of the country’s four major woody oil plants, alongside tea oil, walnut, and Chinese tallow trees [29]. With a cultivation history spanning over a thousand years, the tung oil tree is primarily distributed across several provinces in China, including Yunnan, Guangxi, Jiangxi, Hunan, Jiangsu, and Fujian. The byproduct of tung oil extraction, known as tung cake (or tung bran or tung dregs), is rich in valuable nutrients such as protein, cellulose, amino acids, fatty acids, and sugars [30]. Some of the toxic protein components in tung cake have shown potential as wood adhesives, helping to address issues such as insect infestation. As a result, tung cake is considered an excellent environmentally friendly resource for industrial-grade adhesives. Studies indicate that China produces over 200,000 tons of tung cake annually. Machine-pressed tung cake contains approximately 25% to 30% protein, while extracted tung cake has a crude protein content ranging from 42% to 45% [31]. Its nutritional value surpasses that of other plant cakes, such as vegetable cake, cotton cake, and tea cake. As the primary purpose of tung oil production is oil extraction, tung cake, a high-quality adhesive raw material, is often discarded as waste in traditional processing methods. As a result, less than 20% of tung cake is currently utilized [32,33]. Given this, it is crucial to explore efficient ways to utilize waste tung cake and transform it into a natural, eco-friendly biomass-based adhesive. Successfully developing and utilizing tung cake can not only reduce the artificial board industry’s dependence on synthetic resin adhesives derived from petrochemical resources but also offer advantages over formaldehyde-based adhesives, such as being more environmentally friendly, renewable, and having a wide range of raw material sources. This is of significant importance for the development of biomass-based wood adhesives and fills the research gap in tung oil cake protein adhesives.

In this study, tung cake sourced from Qiubei County in the Wenshan Zhuang and Miao Autonomous Prefecture of Yunnan Province was used as the primary material. The optimal processing parameters for creating a tung cake protein adhesive were determined based on the addition of sodium hydroxide (NaOH), reaction temperature, and reaction time [34]. To enhance the adhesive properties, potassium permanganate (KMnO_4_) and methyl trimethoxy silane (MTMS) were added, forming a cross-linked, formaldehyde-free, and environmentally friendly tung cake protein adhesive system. The bonding strength of the adhesive was analyzed, alongside X-ray diffraction (XRD), Fourier transform infrared spectroscopy (FTIR), and thermogravimetric analysis (TGA) were performed to identify the ideal process parameters. The reaction process was further examined using additional characterization techniques, including thermogravimetric analysis (TG/TGA), differential scanning calorimetry (DSC), and scanning electron microscopy (SEM). Through these methods, a tung cake protein adhesive was developed that offers a simple production process, excellent performance, and environmentally sustainable properties. This research aims to enhance the economic value of tung cake, providing both theoretical foundations and technical guidance for the future sustainable development of tung cake-based biomass materials.

## 2. Materials and Methods

### 2.1. Materials

The following materials were used: tung cake, industrial grade (protein content 44.6%), from Shuanglong Oil & Fat Co., Ltd., Qiubei County, Wenshan Zhuang and Miao Autonomous Prefecture, Yunnan, China; sodium hydroxide (NaOH), 96.0% analytical grade, from Jinshan Chemical Reagent Co., Ltd., Chengdu, Sichuan, China; potassium permanganate (KMnO_4_), 99.5% analytical grade, from Qihongyuan Technology Co., Ltd., Shenzhen, Guangdong, China; methyl trimethoxy silane (MTMS), 98.0% analytical grade, from Aladdin Biochemical Technology Co., Ltd., Shanghai, China; and poplar wood (*Populus* spp.), poplar veneer (400 mm × 400 mm × 1.8 mm), from Minsheng Wood Industry, Linyi, Shandong, China.

### 2.2. Methods

#### 2.2.1. Preparation Process of Tung Cake Protein Adhesive

**Figure 1:**
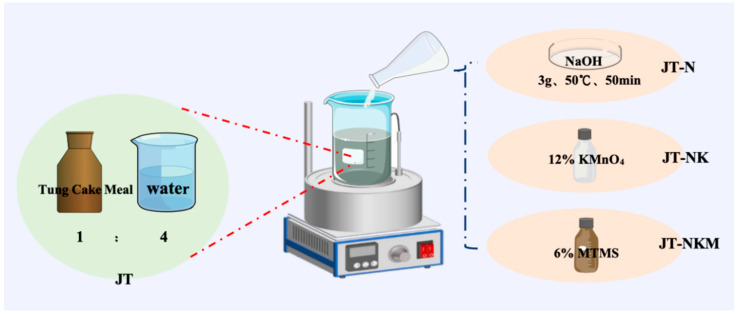
Preparation technology for tung cake protein adhesive.

The preparation process of tung cake protein adhesive is illustrated in Figure 1. A single-factor experimental design was employed, where tung cake and deionized water were mixed in a 1:4 ratio (i.e., unmodified tung cake protein, JT). The experiment was divided into two parts: (1) Alkali treatment (JT-N): The first step involved determining the optimal process parameters for the tung cake protein adhesive, specifically the amount of NaOH added, reaction temperature, and reaction time [35]. During this process, the tung cake protein underwent alkali treatment, which unrolled and relaxed the protein structure, facilitating modification. (2) Oxidative and cross-linking modification (JT-NK and JT-NKM): In the second step, potassium permanganate (KMnO_4_) was added to oxidatively modify the alkali-treated tung cake protein (JT-NK). Following this, methyl trimethoxy silane (MTMS) was introduced for cross-linking modification (JT-NKM), which formed a spatial network structure, enhancing the bonding properties of the adhesive [36]. The system was stirred until a homogeneous solution was obtained, resulting in a light brown, viscous adhesive. This final product was the tung cake protein adhesive.

#### 2.2.2. Viscosity Test

The viscosity of the JT-N tung cake protein adhesive was measured using an NDJ-8S digital viscometer over a shear rate range of 0 to 300 s^−1^. Following the guidelines set by GB/T 14074-2017 (Test Methods for Adhesives and Resins in the Wood Industry), each sample was tested five times, and the average value of the viscosity was recorded to ensure the accuracy and reliability of the results [37].

#### 2.2.3. Preparation of 3-Layer Plywood and Evaluation of Gluing Properties

Three-layer plywood was prepared using tung cake protein adhesives, including JT-NK and JT-NKM, following the specified process. The adhesive was applied at a rate of 360 g/m^2^ on both sides of the veneer. The hot-pressing conditions were as follows: 160 °C temperature, 6 min pressing time, and 1.5 MPa pressure [38]. The gluing properties of the plywood were evaluated according to GB/T 17657-2022 (Test Methods for Physical and Chemical Properties of Wood-Based Panels and Veneered Wood-Based Panels) [39]. Bonding strength was tested using a universal mechanical testing machine (Shimadzu Corporation AG-50KN, Kyoto City, Japan). The following tests were conducted: dry bonding strength, wet bonding strength after immersion at 20 °C for 3 h, and wet bonding strength under hot water conditions after immersion at 63 °C for 3 h. Each sample was tested in triplicate, and the average value was recorded for each test.

#### 2.2.4. X-Ray Diffraction (XRD) Analysis

Four cured tung cake protein adhesive samples, JT, JT-N, JT-NK, and JT-NKM, were analyzed using an Ultima IV X-ray Diffraction (XRD) Analyzer (Rigaku Corporation, Kyoto City, Japan). The solid adhesive samples were first ground into fine powders using an agate mortar and then passed through a 200-mesh sieve. The powdered samples were analyzed by XRD at room temperature with X-rays of wavelength λ = 0.15406 nm. The XRD patterns were recorded over a scanning range of 10° to 80° in terms of the 2θ angle, using a scanning step frequency of 10°/min.

#### 2.2.5. Fourier Transform Infrared Spectroscopy (FTIR) Analysis

The infrared spectra of four tung cake protein adhesives, JT, JT-N, JT-NK, and JT-NKM, were recorded using an iS50 Fourier Transform Infrared Spectrometer (Instron Corporation, Norwood, MA, USA). The adhesive samples were first cured and dried in a 120 °C forced-air drying oven until they reached a constant weight. The samples were then prepared using the potassium bromide (KBr) tableting method, ground into powder (passed through a 200-mesh sieve), and analyzed in the wavelength range of 400–4000 cm^−1^ with 32 scans per sample.

#### 2.2.6. Thermogravimetric Analysis (TG/TGA)

The thermal stability of four cured tung cake protein adhesive samples, JT, JT-N, JT-NK, and JT-NKM, was evaluated using thermogravimetric analysis (Netzsch TG 209 F1, Bavaria, Germany). The samples were heated from room temperature to 600 °C, and the weight loss was measured to assess their thermal stability and decomposition characteristics.

#### 2.2.7. Differential Scanning Calorimetry (DSC) Analysis

Four cured tung cake protein adhesive samples, JT, JT-N, JT-NK, and JT-NKM, were analyzed using a DSC 204 Differential Scanning Calorimeter (Netzsch, Bavaria, Germany). The samples were heated in the temperature range of 0 to 250 °C under nitrogen protection, with a heating rate of 15 K/min. This analysis provided insights into the thermal transitions and curing behavior of the adhesives.

#### 2.2.8. Scanning Electron Microscopy (SEM) Analysis

The cross-sectional structural morphology of four cured tung cake protein adhesive samples, JT, JT-N, JT-NK, and JT-NKM, was observed and analyzed using a Hitachi Regulus 8100 scanning electron microscope (Hitachi High-Tech Science Corporation, Beijing, China). The adhesive samples were dried in an oven at 103 °C for 12 h. After drying, the cross-sectional surfaces of the cured adhesive samples were coated with gold, and their microscopic morphology was examined under the electron microscope.

## 3. Results

### 3.1. Alkali-Modified Viscosity Analysis

**Figure 2:**
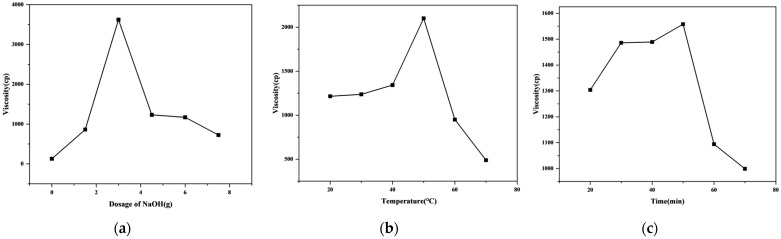
(**a**) Effect of NaOH concentration on the viscosity of JT-N adhesive; (**b**) effect of NaOH reaction temperature on the viscosity of JT-N adhesive; (**c**) effect of NaOH reaction time on the viscosity of JT-N adhesive.

As an innovative biomass-based adhesive, tung cake protein adhesive is rich in polar functional groups inherent to the tung cake protein. These groups, when subjected to alkali treatment, are liberated through depolymerization, influencing the adhesive’s cross-linking density and ultimately its performance [40,41]. The optimal parameters for alkali modification in the preparation of JT-N adhesive were determined by varying the concentrations of NaOH, the reaction temperature, and the reaction time [42], as illustrated in Figure 2a–c. As illustrated in the figure, it is evident that the viscosity of the JT-N adhesive exhibits an initial increase followed by a decline. Under a reaction temperature of 60 °C and a reaction time of 60 min (Figure 2a), the viscosity reaches its peak value of 3623 cP when 3 g NaOH (30%) is added. When the reaction temperature is 50 °C with the same NaOH concentration and a reaction time of 60 min (Figure 2b), the viscosity peaks at 2101 cP. Similarly, when the reaction time is extended to 50 min at 50 °C with 3 g NaOH (Figure 2c), the viscosity achieves a maximum of 1558 cP. This observed trend can be attributed to the progressive penetration of NaOH into the coiled structure of the tung cake protein, which causes the molecular chains to gradually unwind and relax, thereby enhancing the viscosity [43]. However, beyond a certain threshold—whether in terms of excessive NaOH concentration, elevated reaction temperature, or prolonged reaction time—the tung cake protein molecules degrade, reducing their cross-linking potential and diminishing the viscosity [44,45,46]. Through comprehensive analysis, it was determined that the optimal process parameters for JT-N adhesive are 30% NaOH, 50 °C, and 50 min. These conditions not only offer a sound foundation for the current formulation but also pave the way for further refinements and enhancements in subsequent modifications.

### 3.2. Analysis of Bonding Strength Enhancement by Cross-Linking Modification

**Figure 3:**
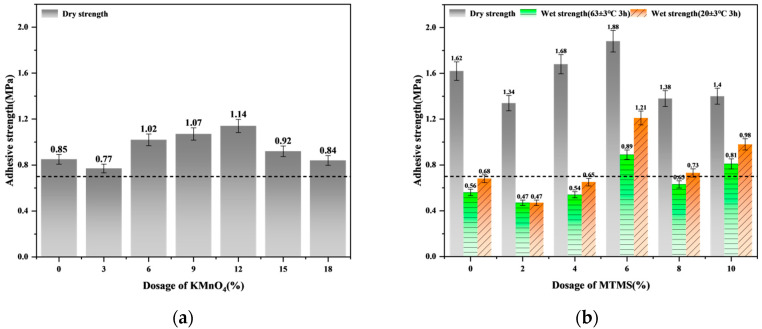
(**a**) Effect of KMnO₄ addition on the bonding strength of tung cake protein adhesive; (**b**) effect of MTMS addition on the bonding strength of tung cake protein adhesive.

Based on the alkali treatment parameters established for the tung cake protein adhesive, the bonding strengths of JT-NK and JT-NKM adhesives, enhanced by cross-linking modification, were measured and are illustrated in Figure 3. In Figure 3a, the dry strength of the JT-N adhesive is shown to be 0.85 MPa. With the addition of KMnO_4_, the dry strength of the JT-NK adhesive peaks at 1.14 MPa with a 12% KMnO_4_ addition—an increase of 34.12%. However, this formulation still lacks water resistance, displaying no measurable wet strength. Figure 3b shows that incorporating MTMS into the formulation substantially improves both dry and wet bonding strengths for JT-NKM adhesive. At a 6% MTMS addition, the dry strength, 20 °C cold water wet strength, and 63 °C warm water wet strength reach maximum values of 1.88 MPa, 1.21 MPa, and 0.89 MPa, respectively. These represent increases of 16.77%, 70.42%, and 67.92%, meeting the Class II plywood standard specified in GB/T 17657-2022 (minimum requirement: >0.7 MPa).

These results indicate that NaOH and KMnO_4_ modifications facilitate the depolymerization of tung cake protein, releasing polar groups previously encapsulated within the protein’s spherical structure [47,48]. This release enhances protein–wood adsorption, significantly increasing bonding strength. When further modified with MTMS as a cross-linking agent, siloxy groups hydrolyze and condense with the protein’s hydroxyl groups, establishing robust chemical bonds. This process forms a spatial cross-linked network, thereby substantially improving the adhesive’s water-resistant bonding performance. Thus, it improves the durability and resistance to moisture and heat aging of the adhesive [49]. However, when an excessive amount of MTMS is added, the surplus alkoxy groups hinder the completion of the reaction, leading to an overproduction of silanol groups [22]. This, in turn, increases the distance between the adhesive’s molecular chains, thereby enhancing relative slippage. Additionally, the excess coupling agent is unable to effectively integrate with the molecular chain to form a proper cross-linking structure, preventing further increases in cross-linking density. In fact, the surplus coupling agent may disrupt the existing cross-linking network, ultimately diminishing the adhesive’s bonding performance [50,51]. In summary, the optimal preparation parameters for tung cake protein adhesive are 30% NaOH (50 °C, 50 min), 12% KMnO_4_, and 6% MTMS, yielding an adhesive with optimal bonding strength.

### 3.3. XRD Analysis

**Figure 4:**
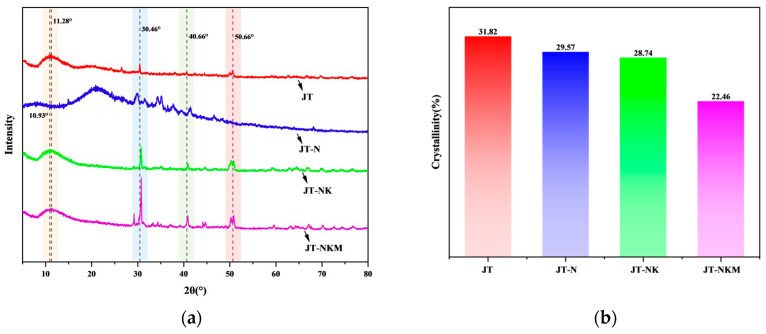
(**a**) XRD curves of tung cake protein adhesives; (**b**) the crystallinity diagram of tung cake protein adhesives.

X-ray diffraction (XRD) was used to analyze the crystallinity of the crystalline regions in tung cake protein adhesives, including JT, JT-N, JT-NK, and JT-NKM, as shown in Figure 4. As seen in Figure 4a, the XRD spectrum of the tung cake protein adhesive displays four prominent diffraction peaks at 2θ = 11.28°, 30.46°, 40.66°, and 50.66°, respectively. Notably, the diffraction peak of the JT-NKM adhesive, modified with KMnO_4_ and MTMS, shifts from 2θ = 11.28° to 10.93°, indicating a change in the protein’s crystalline structure [36,52]. This shift is attributed to the oxidation of the active groups on the tung cake protein molecular chain and their subsequent cross-linking with silane [53]. As shown in Figure 4b, the crystallinity of the JT-NKM adhesive is the lowest at 22.46%, confirming that the addition of KMnO_4_ and MTMS facilitates the cross-linking of the active groups on the tung cake protein molecules, resulting in the formation of a dense three-dimensional network structure [54]. This structure enhances water resistance and consequently improves the adhesive’s bonding performance.

### 3.4. FTIR Analysis

**Figure 5:**
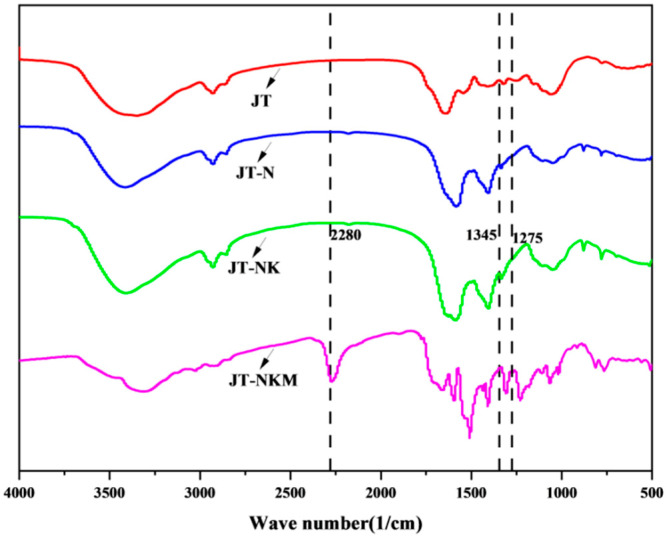
FTIR curves of tung cake protein adhesives.

Fourier transform infrared spectroscopy (FTIR) was employed to investigate the thermal curing properties of tung cake protein adhesives, including JT, JT-N, JT-NK, and JT-NKM, as illustrated in Figure 5. As depicted in Figure 5, the absorption peak at 2280 cm^−^¹ corresponds to the stretching vibration of the Si-H group. With an increase in the amount of the cross-linking agent MTMS, the intensity of this vibration peak is markedly enhanced, suggesting that the silicon–hydrogen bond undergoes an addition reaction to form a co-modified silicone oil, thereby significantly improving the water-resistant bonding performance of the tung cake protein adhesive. Concurrently, the absorption peak at 1275 cm^−^¹, representing the bending vibration of the N-H group, diminishes in intensity with the rising concentration of MTMS [55,56]. This attenuation indicates that the aldehyde group, formed through the oxidation of the sugar carbohydrates in the tung cake, reacts with the amino groups in the protein, leading to the consumption of the amino groups and a reduction in the hydrophilic groups. This, in turn, results in a marked improvement in the water resistance of the JT-NKM adhesive. Similarly, the absorption peak at 1345 cm^−^¹, characteristic of the stretching vibration of the C-N group, exhibits a pronounced increase in intensity and peak area compared to those of the JT and JT-N curves. This enhancement provides compelling evidence that the aldehyde group formed through oxidation reacts with the amino groups in the protein to form C-N bonds, thereby confirming the occurrence of a robust cross-linking reaction between the tung cake protein and silane [57]. This reaction aligns with the increased bonding strength observed in previous studies, thus fulfilling the objective of enhancing the adhesive’s performance.

### 3.5. TG/TGA and DSC Analysis

**Figure 6:**
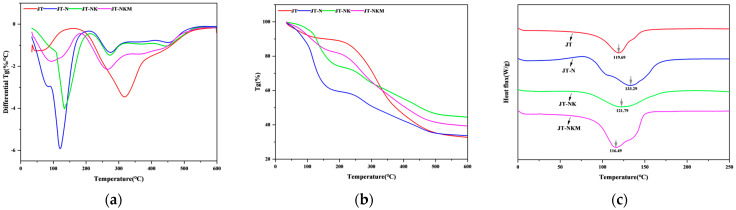
(**a**,**b**) TG/TGA curves of tung cake protein adhesives; (**c**) DSC curves of tung cake protein adhesives.

Thermogravimetric analysis (TG/TGA) and differential scanning calorimetry (DSC) were employed to investigate the thermal stability and thermal curing properties of tung cake protein adhesives, including JT, JT-N, JT-NK, and JT-NKM, as illustrated in Figure 6. As depicted in Figure 6a,b, the thermal degradation process of the tung cake protein adhesives can be divided into two distinct stages. The first stage, spanning from room temperature to 210 °C, primarily involves the complete evaporation of water from the adhesive, followed by the onset of the first weight loss rate peak, which corresponds to the degradation of small molecular compounds within the adhesive [58]. During this stage, the decomposition rates of the JT-N, JT-NK, and JT-NKM adhesives are lower than that of JT, indicating that the thermal stability of the JT adhesive is relatively poor. The incorporation of NaOH, KMnO_4_, and MTMS effectively enhances the thermal stability of the JT adhesive. The second stage, between 210 °C and 500 °C, primarily involves the partial degradation of tung cake protein molecules, the structural separation of protein molecules, and the cleavage of covalent bonds between amino acid residues [4,43]. In this stage, the decomposition rate of the modified tung cake protein adhesives follows the order JT-N > JT-NK > JT-NKM, with the decomposition rate progressively decreasing and thermal stability significantly improving. This is because MTMS reacts with the hydroxyl groups in the protein adhesive, forming stable siloxane bonds, which in turn enhance the adhesive’s water resistance and durability [59,60]. Moreover, the thermal weight loss of the JT-NKM adhesive is relatively small, suggesting that its curing performance has been notably enhanced.

As shown in Figure 6c, the exothermic temperature of the JT-NK and JT-NKM adhesives is considerably lower than that of the JT-N adhesive (133.29 °C). This reduction is attributed to the cross-linking reaction between KMnO_4_, MTMS, and protein molecules, which accelerates the curing process of the tung cake protein adhesive [61]. Additionally, the curing exothermic peak of the JT-NKM adhesive is the most pronounced, with a larger integral area of the exothermic peak, indicating a higher degree of curing [62]. This implies that the curing reaction of the tung cake protein adhesive is more thorough after treatment. Consequently, under identical hot-pressing conditions, the JT-NKM adhesive exhibits superior curing performance, which correlates with its enhanced water resistance.

### 3.6. SEM Analysis

**Figure 7:**
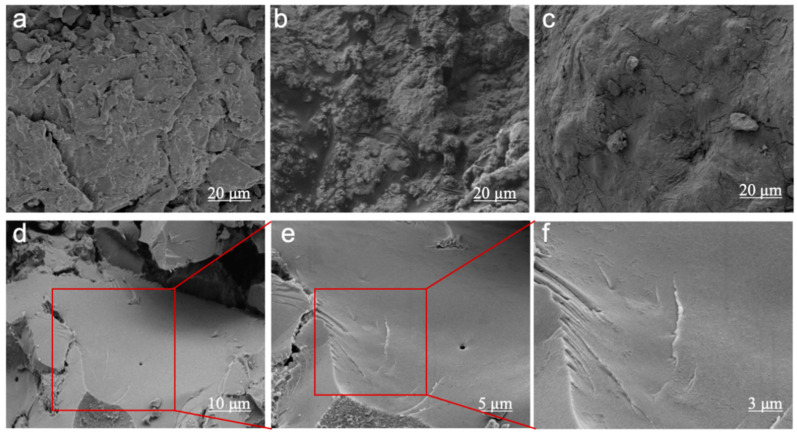
SEM micrograph of tung cake protein adhesives. (**a**): JT adhesive; (**b**): JT-N adhesive; (**c**): JT-NK adhesive; (**d**): 1.00 KX JT-NKM adhesive; (**e**): 2.00 KX JT-NKM adhesive; (**f**): 3.00 KX JT-NKM adhesive.

Scanning electron microscopy (SEM) was employed to characterize the microscopic morphology of tung cake protein adhesives, including JT, JT-N, JT-NK, and JT-NKM, as depicted in Figure 7. As shown in Figure 7a, the JT adhesive reveals numerous irregular spherical shapes under 400 X magnification. Given that the protein content of the tung cake used in this study is 44.6%, with globulin being the primary component of tung cake protein [63], the irregular spherical structures observed in the electron microscope images correspond to the intact tung cake protein. Upon the addition of NaOH (Figure 7b), the cross-sectional morphology of the JT-N adhesive becomes rough and porous. This is attributed to the internal stresses within the adhesive that arise from the evaporation of water during the curing process. Such characteristics indicate that the JT-N adhesive is relatively brittle [64], with the presence of pores providing space for internal moisture, thereby reducing its water resistance. Following the incorporation of KMnO_4_ (Figure 7c), the cross-section of the JT-NK adhesive appears smoother, though numerous particles remain, and a hill-like morphology emerges. This suggests that the oxidation reaction promotes the rapid release of adhesive energy, leading to the refinement of particles. Simultaneously, the spatial configuration of the tung cake protein is altered, presenting a more open, chain-like α-helix or β-folded structure [32,65]. The hill-like morphology observed in the image corresponds to this chain-like arrangement, though the water resistance remains relatively low. Upon the addition of MTMS (Figure 7d,e), the fracture surface of the JT-NKM adhesive becomes markedly smoother, exhibiting a tighter fracture profile and an irregular morphology characterized by ridges and depressions, indicating improved toughness. In the 3.00 KX image (Figure 7f), the cross-section reveals a uniformly dense structure, suggesting that the JT-NK adhesive underwent a reaction with silane, which filled the secondary structure of the protein that was previously unfolded by KMnO_4_. This interaction plays an energy-dissipating role under stress [66], resulting in a more compact cross-section and a smoother surface. The enhanced compactness significantly improves the cross-linking density of the adhesive, confirming the formation of a robust three-dimensional network structure, which in turn imparts superior toughness to the cured adhesive layer. The irregular morphology of ridges and depressions in the image reflects this network structure.

The results demonstrate that the density of the modified tung cake protein adhesives follows the order JT-NKM > JT-NK > JT-N > JT, indicating that JT-NKM exhibits the best water resistance, thereby effectively enhancing both the bonding strength and water resistance of the adhesive. SEM analysis further corroborates that the curing and reaction mechanisms of the tung cake protein adhesive are consistent with the findings from previous bonding strength determinations, as well as the XRD, FTIR, TG/TGA, and DSC analyses.

### 3.7. Strategic Analysis of Cost Efficiency

To further underscore the practical significance of this study, a cost comparison was performed between the tung cake protein adhesive developed herein and the prevailing industrial wood protein adhesives, as presented in Table 1. Upon calculation, the production cost of the tung cake protein adhesive was found to be approximately CNY 2732.8 per ton, which is substantially lower than the market price of comparable products, which stands at CNY 2767.2 per ton—representing a remarkable cost reduction of 50.31%. These findings clearly highlight the significant cost advantages of the tung cake protein adhesive produced in this study.

## 4. Conclusions

This study presents an efficient approach to recycling waste tung cake by optimizing the alkali treatment process through the careful control of sodium hydroxide (NaOH) concentration, reaction temperature, and reaction time. Additionally, the introduction of potassium permanganate (KMnO_4_) and methyl trimethoxy silane (MTMS) serves to cross-link and modify the tung cake protein, resulting in the development of a formaldehyde-free adhesive that is not only simple to produce but also exhibits excellent performance and adheres to green, environmentally friendly principles. The underlying mechanism of this preparation process is as follows: initially, NaOH penetrates the coiled structure of the tung cake protein molecules, causing them to progressively unwind and relax, thereby enhancing the viscosity. Subsequently, KMnO_4_ is introduced to disrupt the secondary protein structure while simultaneously oxidizing the saccharide components in the tung cake. This oxidation reaction with the protein’s amino groups reduces the hydrophilic groups and improves the adhesive’s water resistance. Finally, MTMS is incorporated to cross-link the active sites in the tung cake protein, forming a three-dimensional network structure that enhances thermal stability, reduces weight loss during thermogravimetric analysis, improves curing performance, and significantly boosts bonding strength. As a result, the dry strength, cold water wet strength at 20 °C, and warm water wet strength at 63 °C of the tung cake protein adhesive show considerable improvements. These values surpass the Class II plywood standards outlined in GB/T 17657-2022 (i.e., >0.7), thereby meeting the wood industry’s growing demand for environmentally sustainable wood adhesives.

In conclusion, tung cake protein adhesive, as a novel and sustainable biomass-based wood adhesive, offers several significant advantages over other formaldehyde-free biomass-based adhesives. First, the raw material source is abundant. Tung cake is derived from the residue of tung oil plants, making it not only readily available but also cost-effective, positioning it as a sustainable and economical alternative to traditional adhesives. Unlike synthetic adhesives that depend on petrochemical resources, it benefits from a broader range of raw material sources. Second, its bonding performance is impressive. Tung cake protein adhesive exhibits excellent bonding strength. After modification, it forms a strong cross-linking network, improving water resistance, durability, and long-term stability. Third, the inherent anti-moth properties of tung cake open up exciting possibilities for future research and applications of tung cake protein adhesive, further enhancing its appeal. Therefore, tung cake protein adhesive stands out for its environmental friendliness, economic viability, and superior bonding performance, making it a promising candidate among formaldehyde-free biomass-based adhesives. Its use will not only support the economic value of tung oil trees but also contribute to the efficient utilization and sustainable development of tung cake biomass forest products. This has significant practical implications for the large-scale industrial application of tung cake protein adhesives and will help fill a critical research gap regarding natural materials like tung cake.

## Figures and Tables

**Table 1:** Cost of each component in the tung cake protein adhesive.

Adhesives	Tung Cake Protein Adhesive					Market for Wood-Based Protein Adhesives
Primary materials	Tung cake	Water	NaOH	KMnO_4_	MTMS	-
Price per unit (CNY per ton)	1500	2.80	2000	3000	4500	-
Overall sum (CNY per ton)	2732.80					5500

## Data Availability

All the data are provided in the manuscript.
